# E-textile based modular sEMG suit for large area level of effort analysis

**DOI:** 10.1038/s41598-022-13701-4

**Published:** 2022-06-10

**Authors:** Korine A. Ohiri, Connor O. Pyles, Leslie H. Hamilton, Megan M. Baker, Matthew T. McGuire, Eric Q. Nguyen, Luke E. Osborn, Katelyn M. Rossick, Emil G. McDowell, Leah M. Strohsnitter, Luke J. Currano

**Affiliations:** 1 Research and Exploratory Development Department, The Johns Hopkins Applied Physics Laboratory, Laurel, MD 20723 USA; 2 Air and Missile Defense Sector, The Johns Hopkins Applied Physics Laboratory, Laurel, MD 20723 USA

**Keywords:** Biomedical engineering, Sensors and biosensors

## Abstract

We present a novel design for an e-textile based surface electromyography (sEMG) suit that incorporates stretchable conductive textiles as electrodes and interconnects within an athletic compression garment. The fabrication and assembly approach is a facile combination of laser cutting and heat-press lamination that provides for rapid prototyping of designs in a typical research environment without need for any specialized textile or garment manufacturing equipment. The materials used are robust to wear, resilient to the high strains encountered in clothing, and can be machine laundered. The suit produces sEMG signal quality comparable to conventional adhesive electrodes, but with improved comfort, longevity, and reusability. The embedded electronics provide signal conditioning, amplification, digitization, and processing power to convert the raw EMG signals to a level-of-effort estimation for flexion and extension of the elbow and knee joints. The approach we detail herein is also expected to be extensible to a variety of other electrophysiological sensors.

## Introduction

Bio-potential signal monitoring of muscle firing, generally referred to as electromyography (EMG), enables dynamic, rapid sensing and reporting of the location and intensity of movements in the human body. This powerful technique has been used for applications such as prosthesis control^1–3^, health monitoring^4–6^, and transparent human–machine interfaces^7–9^. While there are invasive forms of EMG, non-invasive surface electromyography (sEMG) is more common and accepted for most applications. The current gold standard for sEMG involves manually attaching tethered, temporary gel electrodes to the skin using an adhesive. While this technology is well established and yields high fidelity signals, its reliance on conductive gels and bulky data acquisition systems degrades its operational lifetime, comfort, and practicality for longitudinal monitoring. As such, there is a need to develop laboratory quality sEMG sensors that are ergonomically integrated into high performance garments.

To address this, significant research efforts have gone into the design and optimization of wearable garments that exploit the use of electronic textiles (e-textiles). In contrast to silver–silver chloride (Ag–AgCl) based systems, these wearable sEMG garments are hybrid circuits that incorporate flexible e-textile based electrodes and interconnects^10–14^. Numerous techniques exist to incorporate conductive materials into a garment including embroidery of conductive fibers^15–19^, printing of conductive inks^20–25^, and using adhesives to attach conductive fabrics to the textile^26^. Of these well-established techniques, embroidery and printing are the most commonly employed. However, they require specialized skills to generate complex, strain relieving patterns and tools specific to the garment industry that are not readily accessible to the average research grade laboratory. Few studies present large area fully integrated systems with end-to-end optimization for quality and manufacturability. Noteworthy exceptions to this are targeted commercial platforms with opaque, self-contained systems that are both expensive and block access to raw, unprocessed data. In contrast, our adhesive-based approach centers on hot lamination to attach conductive fabrics to the base textile, a much simpler approach to garment construction. Furthermore, as demonstrated in this work, this approach can be seamlessly extended to form a facile textile to circuit board connection with flexible printed circuit board tabs for signal transmission to on-board electronics.

Accordingly, to democratize high quality, large area sEMG suits, in this work we present a robust, scalable, and fully integrated e-textile based modular sEMG garment made from readily available commercial materials. We perform careful materials and design optimization for each component, characterize electrical performance during strain and exposure, and quantify level of effort measurements for the bicep/tricep, quadriceps/hamstring, and tibialis anterior/gastrocnemius muscles. Beyond our immediate interest of textile-based sEMG, our approach creates a pathway to integrate high fidelity skin electrodes and interconnects for a variety of physiological sensors, including electrocardiogram (ECG), electroencephalography (EEG), and galvanic skin response (GSR).

## Results and discussion

**Figure 1:**
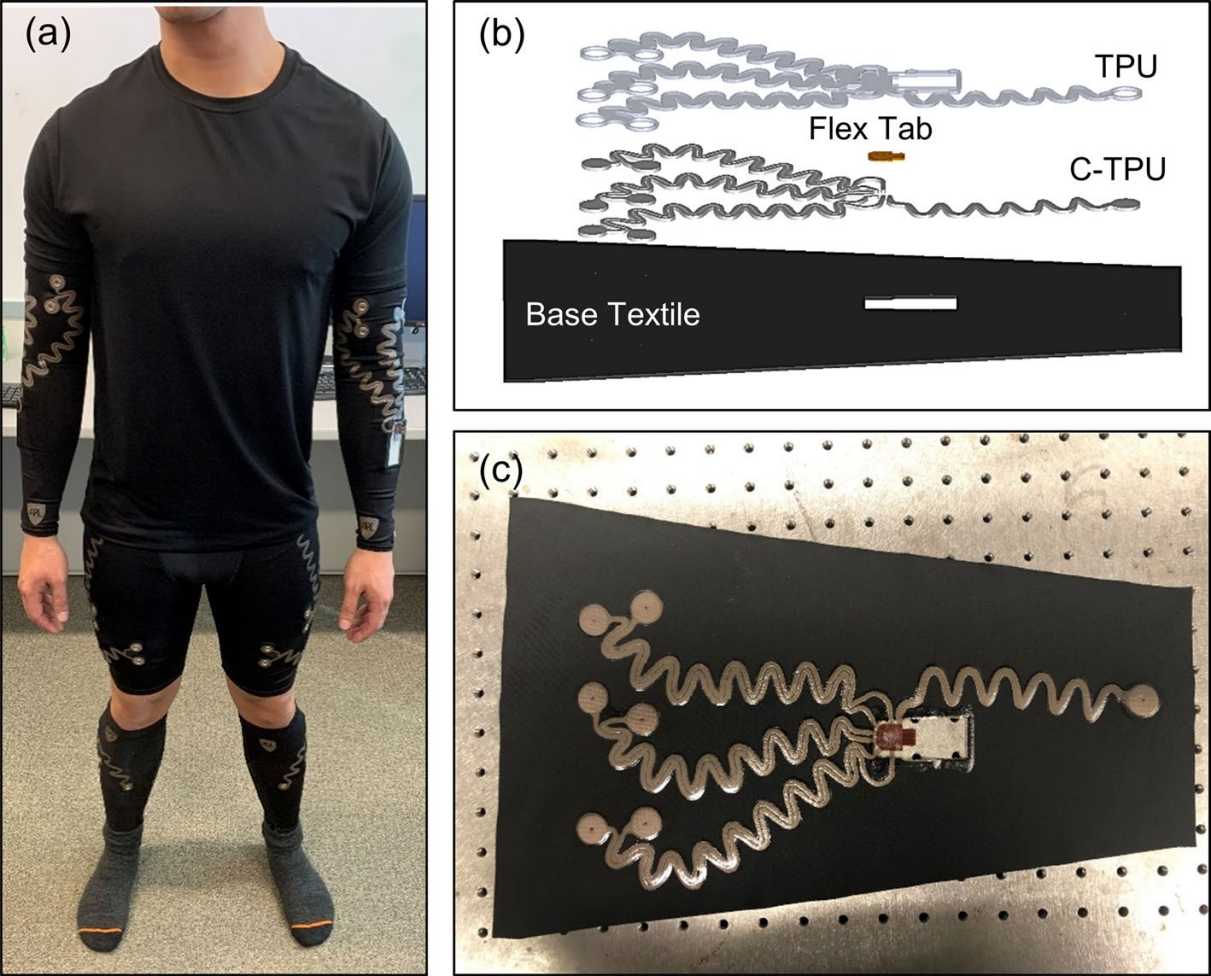
(**a**) Subject wearing the final sEMG suit showing arm sleeves, shorts, and calf sleeves. Fabrication of top side of the sEMG sleeves showing an (**b**) exploded view of sleeve stack up and (**c**) image of the assembled sleeve.

Our modular sEMG suit is comprised of arm sleeves, shorts, and calf sleeves in order to access the bicep/tricep, quadriceps/hamstring, and tibialis anterior/gastrocnemius muscles (Fig. 1a). Each component of the suit was fabricated using the same materials and procedure, with variations only in the pattern of the base fabric and overlaying materials. The base composition of the arm sleeve is shown in Fig. 1b,c below, detailing the final top side configuration. The exterior of the sEMG sleeve was composed of four main layers: (i) a non-conductive high performance athletic base textile (see Supplementary Information), (ii) a patterned conductive layer, (iii) a flexible tab, and (iv) an adhesive backed protective thermoplastic polyurethane layer (Fig. 1b).

The exterior surface of the assembled sleeve is shown in Fig. 1b, before sewing the sleeve closed. Each layer served a functional purpose. The base textile provided compression for improved contact with the skin, sweat wicking, and range of motion. This fabric can readily be cut into generic pre-defined dimensions corresponding to a medium or large size that both have trapezoidal shapes with a length of 47 cm. The large sleeve was approximately 28 cm wide at the top, 1.4 cm wider than the medium sleeve. The conductive layer (C-TPU) served as an interconnect for signal routing from the electrodes to the downstream printed circuit board (PCB). A flexible tab provided textile-to-PCB interconnection through a locking flexible printed circuit (FPC) connector mounted on the PCB. Finally, the overlaid TPU film provided electrical isolation as well as abrasion and moisture protection for the conductive layers.

### Interconnect optimization

To optimize the performance of the interconnects, we characterized the robustness to strain across various materials, treatments, and configurations for the arm sleeve. First, we down-selected the material and assembly configuration by comparing the performance of arm sleeves made using screen printed stretchable silver ink (PE874, DuPont Intexar) or a silver plated lycra based commercial conductive stretch material (CCSM) (A321, Less EMF) for the patterned conductive layer.

**Figure 2:**
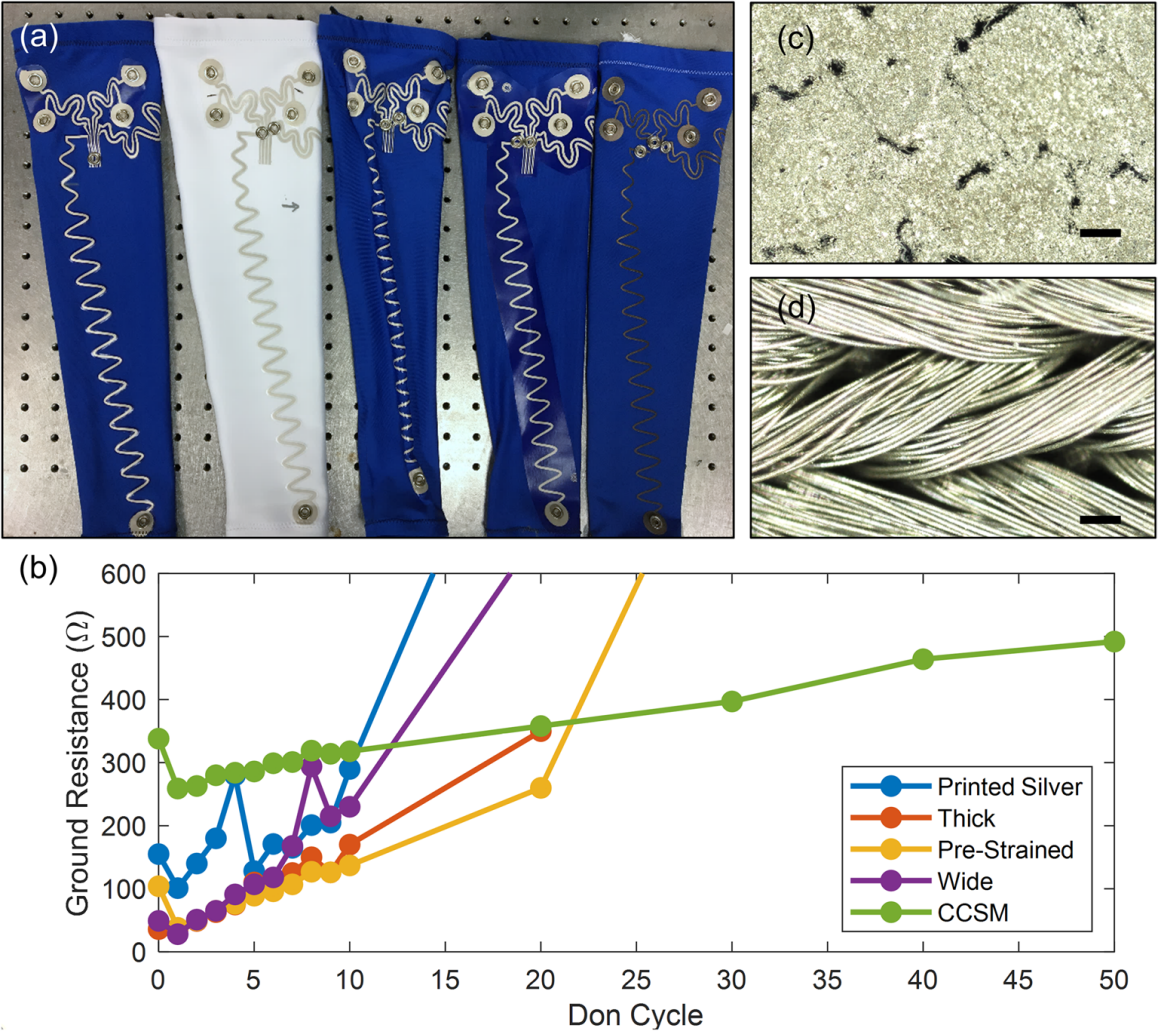
Preliminary don-doff strain testing of completed sleeves showing. (**a**) Image of sleeves showing the printed silver, thick, pre-strained, wide, and CCSM configurations (from left to right). (**b**) Ground resistance below 600 Ω versus don cycle. Bright field optical microscope images of (**c**) printed silver after strain and (**d**) CCSM under strain. Scale bar represents 100 µm.

We tested five configurations: printed silver on our baseline athletic fabric, printed silver on thicker, stiffer high-compression fabric, printed silver on pre-strained athletic fabric, printed silver on wide-cut TPU on athletic fabric, and laser-cut CCSM on athletic fabric (Fig. 2a). The CCSM and printed silver on athletic fabric had the same base geometry to enable direct comparison between the two conductive materials evaluated. Each additional treatment used in conjunction with the screen printed silver ink was selected for potential further strain mitigation, as preliminary testing of the silver ink showed dramatic increases in resistance with repeated strain cycles. The thick rigid fabric was selected to limit the maximum amount of strain exerted on the interconnects during don-doff. The pre-strained fabric was selected as a means to incorporate strain relief into the base fabric. Finally, the wide TPU was selected to selectively stiffen only the region of the base fabric that the interconnects traverse.

To compare these sleeves, we measured the end-to-end resistance of the longest interconnect (which happened to be the ground interconnect on the arm sleeve) while worn by a stationary test subject after a progressively increasing number of don-doff cycles. Sleeves were methodically donned by pulling along the top armband and doffed by tugging from the bottom to mimic the expected complex high strain environment during don-doff. The ground interconnect for each sleeve had an initial resistance below 400 Ω that increased with each don cycle (Fig. 2b).

The baseline printed silver sleeve showed the most rapid increase, exceeding our self-defined threshold of 600 Ω by 15 don cycles. Following this, resistance of the wide TPU configuration surpassed this threshold by 20 don cycles, and the pre-strained fabric reached this threshold by 30 don cycles. While the thick fabric showed the most promise of the printed interconnects, having a resistance less than 400 Ω by 20 don cycles, it was significantly less comfortable to wear and thus eliminated. Notably, the ground resistance for the CCSM sample remained below the threshold of 600 Ω out to at least 50 don cycles, despite having no further strain mitigation treatments like those used on the printed silver sleeves. These results indicate that while various strain mitigation treatments can slow the resistance creep of the printed silver conductor during don-doff compared to baseline, fundamental differences between the robustness of the conductive materials (i.e. printed silver and CCSM) to strain have a significant impact on their durability. The impressive performance of the CCSM was likely due to the enhanced stretchability of conductive woven materials compared to the screen-printed materials. With CCSM, the conductive layer is plated onto a flexible fabric, resulting in a conductive coating over each individual fiber, whereas screen-printed sleeves relied solely on the adhesion of the ink to the underlying TPU. This prediction was validated upon imaging of each type of sleeve when relaxed and strained. While the screen printed silver formed cracks and defects in the film during strain (Fig. 2c), the pressed CCSM remained tightly woven and readily extended (Fig. 2d). Consequently, we chose CCSM as the conductive material with no additional fabric or TPU treatments to streamline the fabrication procedure.

**Figure 3:**
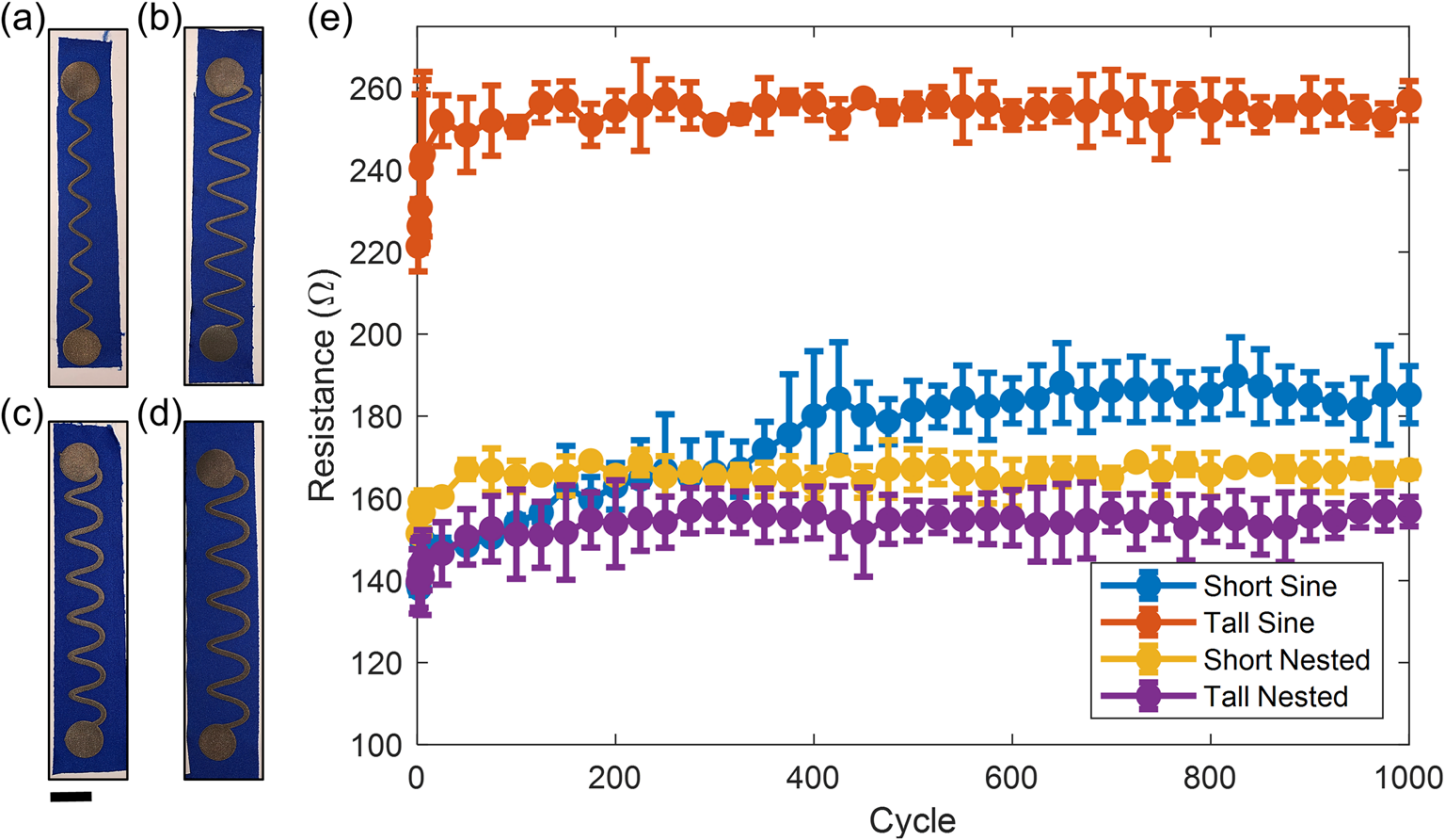
Strain cycling results showing the (**a**) short sine interconnect design, (**b**) tall sine interconnect design, (**c**) short nested interconnect design, and (**d**) tall nested interconnect design. Scale bar represents 2 cm. (**e**) Resistance versus cycle number at 25% strain over 1000 cycles (n = 3 coupons).

Next, we investigated the stability of four different periodic designs during uniaxial strain cycling of the CCSM conductor on swatches of athletic textile. Each design was either a simple sinusoid or a “nested sinusoid” permutation and had a short or tall amplitude (Fig. 3a–d). These designs were chosen due to the known, well-established benefits of meandering patterns as opposed to straight paths for enhanced tolerance to stretching-mode deformations^27,28^. The “nested” design has alternately larger amplitude/period for the first half of each cycle and smaller amplitude/period for the second half. This was designed to accommodate the parallel interconnects needed for differential sEMG channels—the other interconnect of each pair will alternate between smaller amplitude/period and larger amplitude/period to “nest” the two sinusoids together over the interconnect length and conserve space on the sleeve.

The ordinary sinusoid samples had the same 20 mm period, with amplitudes of 19 mm and 10 mm for the tall and short designs, respectively. The tall nested design had a larger period (i.e. 23 mm) and amplitude (i.e. 20 mm) compared to the short design (i.e. 20 mm and 15 mm, respectively), but both had a comparable effective length of approximately 24 cm. To select for optimal robustness over time, we conducted waveform cyclic testing on an instron load frame to an amplitude of 25% strain, at a frequency of 1 Hz, over 1000 strain cycles. Resistance measurements were averaged over three samples for each design (Fig. 3e). For consistency, each test sample had the same physical length (i.e. 13 cm center to center from the contacts) and was mounted on the flexible performance fabric along the same thread orientation.

The tall pure sinusoid design exhibited the highest average resistance across all designs (i.e. ≈ 252 Ω), having a longer effective length compared to the short sinusoid, and smaller cross sectional area compared to the nested designs. Specifically, the tall sin had a length/area of ~ 1.6 × higher than the other test coupon designs, corresponding closely to the ~ 1.6 × higher starting resistance of the tall sin test coupon*.* While the remaining three designs had comparable average resistances, the short pure sinusoid had the worst stability to strain cycling, exhibiting a resistance increase of approximately 33% after 1000 cycles. The nested designs showed moderate stabilities, exhibiting increases of approximately 12% and 10% for the short and tall designs, respectively. Notably, the majority of the resistance increase is in a brief initial settling period for all but the short pure sine. Ultimately, the tall nested design exhibited the lowest average resistance of ≈ 153 Ω. These results showed that the wider nested designs outperform thin pure sinusoids during axial strain. This is consistent with the known geometry dependence of strain relief designs, where at a consistent thickness wide sinusoidal features show enhanced stretchability prior to reaching a critical buckling strain^29^.

**Figure 4:**
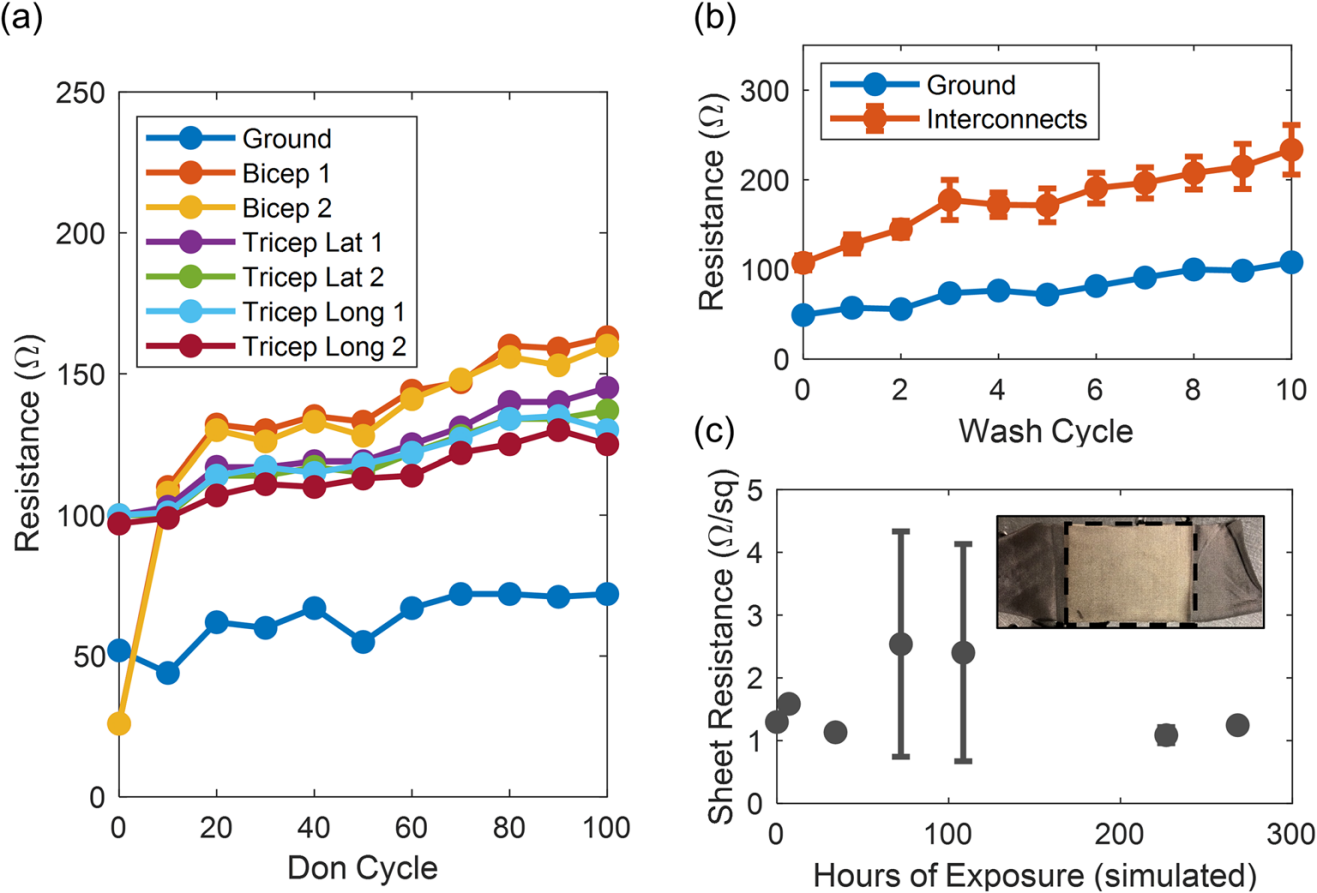
Robustness testing of finalized interconnect geometry showing (**a**) end to end resistance of CCSM leads for each muscle group versus don-doff cycle, (**b**) end to end resistance of CCSM leads versus machine laundering cycles (n = 4 electrode interconnects), and (**c**) sheet resistance of CCSM versus simulated hours of exposure to UV (n = 3 coupons, inset image of sample test coupon with exposed region enclosed).

Following these findings, we modified the design to use the tall nested configuration, and also modified the aspect ratio of the wrist/ground interconnect such that as fabricated, all interconnects had a resistance of 100 Ω or less. We fabricated new arm sleeves and quantified the resistance of each interconnect during the complex strain environment of don-doff and machine washing (Fig. 4a,b). Our findings were consistent with our previous don-doff study of the earlier design, showing a gradual increase in resistance with increasing don-doff cycles. Despite this, the end-to-end resistance in each interconnect remained well below 200 Ω out to the 100th don cycle. Further, consistent with strain cycling results, each interconnect showed relative stability to strain cycling after an initial settling period, having average increases below 20% and 50% by 50th and 100th cycles, respectively.

Additionally, we measured the change in resistance as a result of machine washing (Fig. 4b). By the third wash, both the ground and electrode interconnects exhibited a ≥ 50% increase in resistance. Accordingly, individual washes have a significantly higher impact on strain evolution than individual don-doff cycles. In this more complex environment, accelerated wear occurs, compounding the degradation of the conductive textile^30,31^. Additionally, over the course of 10 washes, this process led to a 120% and 117% increase in resistance for the ground and electrode interconnects, respectively. While not reported here, we expect hand washing to exert less strain and improve the lifetime of the garment further^32^. Additionally, improved robustness to washing can be achieved by coating the electrodes in a flexible hydrophobic film such as polydimethylsiloxane (PDMS)^33^. Nevertheless, CCSM interconnects with a reasonable strain relief design have proven to be quite robust, with the end-to-end resistance increasing less than 1.5 × the original value for both 100 don-doff cycles and 10 machine wash cycles.

As a final means to characterize the durability of the CCSM, we recorded the sheet resistance of test coupons over the course of 268 simulated hours of UV exposure (Fig. 4c). Overall, the impedance of the CCSM test coupons remained roughly constant at a modest average value of 1.6 Ω/sq. While UV exposure led to a noticeable color change, the electrical properties were relatively stable over the duration of the experiment. Thus, CCSM exhibits excellent stability to both UV exposure and strain for large area wearable electronic applications.

### Skin-contact electrode optimization

**Figure 5:**
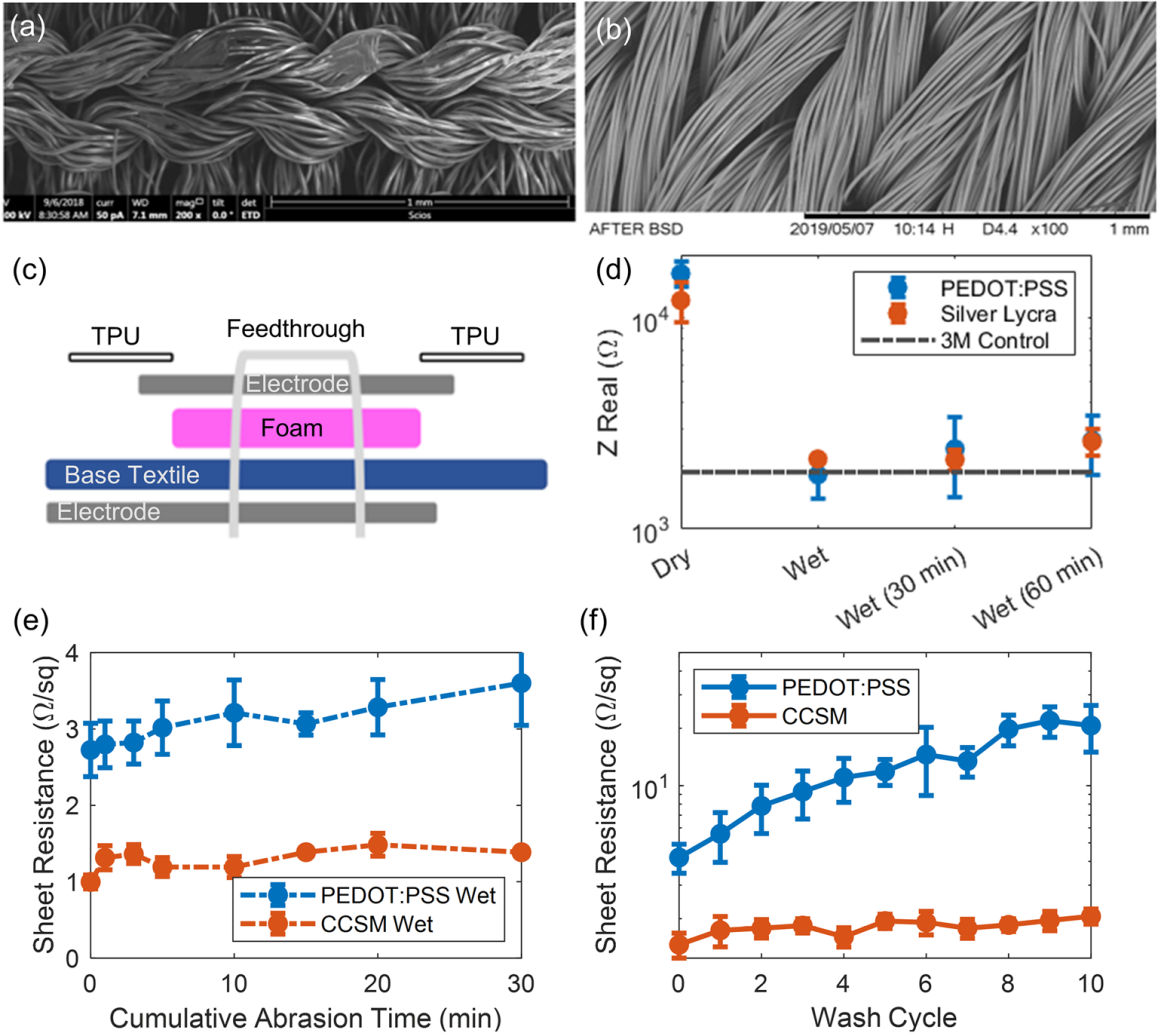
Characterization of PEDOT:PSS and CCSM electrodes. SEM image of (**a**) polyester impregnated with PEDOT:PSS and (**b**) CCSM. (**c**) Schematic of electrode stack up. (**d**) Impedance measurements of PEDOT:PSS and CCSM when dry and wet at 1 kHz (n = 2 coupons). Sheet resistance of PEDOT:PSS and CCSM versus (**e**) cumulative abrasion time (n = 3 coupons) and (**f**) laundering cycles (n = 5 coupons).

We also investigated the viability of research grade poly(3,4-ethylenedioxythiophene) polystyrene sulfonate (PEDOT:PSS) and CCSM materials for use as skin-contact electrodes for electrophysiological measurements. Here, we selected PEDOT:PSS for comparison due to the extensive existing research into this conductive polymer for bio-electrode applications^34^, high conductivity^35^, moisture stability^36^, and air stability^37^. For testing, we impregnated a polyester swatch with PEDOT:PSS solution (Fig. 5a) for comparison with a coupon of commercial CCSM (Fig. 5b). The electrode stack up is comprised of (i) the frontside CCSM contact pad, (ii) base athletic textile, (iii) foam, (iv) the backside conductive electrode material, and a (v) protective layer of TPU (Fig. 5c). Here, the textile electrodes have a foam backer to increase the compression at the sEMG sites and decrease the contact resistance to the skin. Additionally, we include a snap feedthrough for interconnection between the backside electrode material and frontside CCSM contact pad. The TPU layer increases comfort of the garment by protecting the skin from the rough edges of the embedded foam.

First, to compare the baseline performance of PEDOT:PSS and CCSM to state of the art 3 M commercial Ag–AgCl gel electrodes, we measured the impedance when dry, and immediately after immersion in water to see the impact of moisture on the system. We also allowed the coupons to air-dry for 30 min and 60 min and retested them, to explore the duration of the improvement (Fig. 5d). The setup for this experiment used two electrodes in contact with the skin on the bicep of a healthy male volunteer, and impedance between the two electrodes was measured at 1 kHz using a Gamry Reference 3000 Electrochemical Impedance Analyzer system. For the commercial gel electrodes, the electrodes were held in place by the built-in adhesive. For the PEDOT:PSS and CCSM electrodes, the electrodes were attached to fabric coupons and placed in between the skin and a commercial athletic compression sleeve. The baseline impedance of both textile electrode pairs is roughly an order of magnitude larger than the gelled 3 M electrodes, with CCSM having an average impedance 34% lower than PEDOT:PSS. Once hydrated the average impedance of both test coupons dramatically improved, declining by roughly 9 × and 5 × for PEDOT:PSS and CCSM, respectively. Even after 60 min, the textile electrode impedance was still comparable to (though slightly higher than) the 3 M baseline. While overall impedance values for the two textile electrodes were similar, the CCSM impedance was more consistent across multiple trials than the PEDOT:PSS. This is likely due to the more controlled commercial manufacturing process for CCSM versus our lab-produced PEDOT:PSS impregnated textile electrodes.

Next, we characterized the durability of the wet electrode materials to abrasion (Fig. 5e). For these tests, we simulated aggressive abrasion of damp electrode materials (PEDOT:PSS polyester or CCSM) against a secondary cotton cloth with a Taber linear abraser. Testing was performed wet because initial spot checks showed this was produced more wear than dry abrasion, so is the more demanding condition. The 1 kg force and 1 Hz frequency of the experimental apparatus exerts accelerated wear on the test coupon compared to that expected from normal operation to predict extreme conditions. Despite this, both PEDOT:PSS and CCSM test coupons remained well under 4 Ω/sq over the duration of the experiment, having at most 32% and 49% increases from low initial sheet resistances of 2.7 and 1 Ω/sq respectively. Accordingly, both samples performed well and exhibited a minimal increase in sheet resistance. Notably, both CCSM and PEDOT:PSS are percolated networks of fabrics impregnated with conductive functional coatings. Thus, during deformation and handling conductive pathways can persist via hinging/sliding along new intersection points for the individual fibers; explaining the high performance of both materials.

Finally, to characterize the durability of each electrode material to detergent, we measured the sheet resistance across 10 hand wash cycles (Fig. 5f). This mild laundering technique compared to machine washing led to a negligible change in sheet resistance for the CCSM sample (as compared to that reported in Fig. 4b). Over 10 wash cycles, the average sheet resistance of the CCSM consistently remained approximately 1.8 Ω/sq. Contrastingly, despite gentle handling the PEDOT:PSS samples showed a 5 × increase in sheet resistance over 10 wash cycles, indicating poor stability to laundering. This significant degradation in sheet resistance for PEDOT:PSS due to laundering is likely a result of poor adhesion of PEDOT:PSS samples to the underlying fabric compared to the plated CCSM. While abrasion inflicts surface level damage that can be compensated by conductive pathways through underlying unaffected layers, laundering exerts volumetric wear that penetrates to all fibers in the sample. Thus, due to its better consistency, stability, and durability, we ultimately selected CCSM as the skin-contact electrode material for the final garment.

### Final design and level of effort analysis

**Figure 6:**
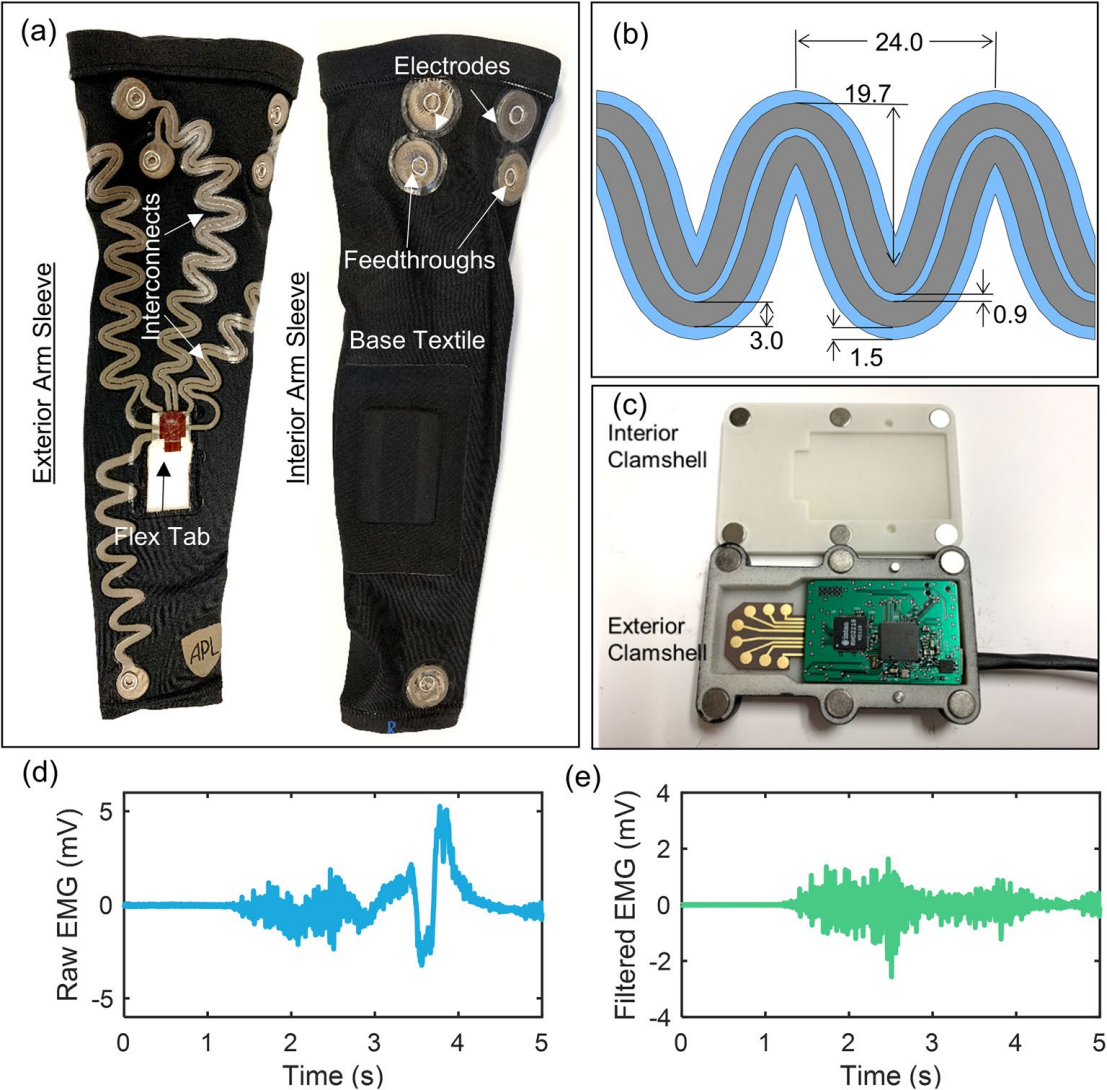
Finalized arm sleeve design showing the (**a**) labeled image of the interior and exterior of the sleeve, (**b**) schematic of tall nested interconnect design showing e-textile (gray/dark) and protective TPU (blue/light, dimensions in mm), and (**c**) image of the flex tab in the optional custom built glass filled nylon enclosure. Exemplar elbow flexion EMG plot from the arm sleeve showing (**d**) raw data and (**e**) filtered data.

The finalized sEMG garment gathered electrical signals produced during muscle activation from the surface of the skin of the wearer, and carried them to a circuit board that amplifies and processes the electrical signals (Fig. 6a). The garment had multiple conductive CCSM based textile electrodes positioned on the inside surface to make contact with the skin over specific muscles and/or muscle groups. To ensure good contact to the skin, the fabric for the garment was cut to size for small, medium, or large individuals and a cushion was included underneath the internal electrode. These cushions added thickness at the measurement site and aided in local compression. Further, the thermoplastic polyurethane surrounding the electrode increases adhesion at the measurement cite to maintain contact during normal activity. With these design enhancements, the CCSM based electrodes exhibited favorable baseline noise and signal to noise ratios in comparison to conventional gel electrodes (see Supplementary Information). Once the sEMG signal is collected, it travels from the inside of the garment to the outside through a metallic snap fastener, then through a conductive textile interconnect (Fig. 6b) that connects the sEMG sites to a single centrally-located site where the circuit board was mounted on the sleeve (Fig. 6c). Here, the outermost light layer of the Fig. 6b details the dimensions for the overlaid protective TPU and the innermost dark layer details the dimensions for the paired e-textile based interconnects. The Kapton flex printed connector was laminated to the sleeve at one end, with electrical pads in contact with the conductive textile interconnects. The other end of the Kapton flex connector was free-hanging and shaped to mate with a locking flexible printed connector (FPC) socket on the custom circuit board.

The custom circuit board used an Intan RHD2216 electrophysiology amplifier chip to amplify and digitize the sEMG signals, and either an embedded Giant Gecko microcontroller (Fig. 6c) or a PC connected to the sleeve via an Intan RHD USB interface board to perform various processing tasks on the digitized data, depending on the experiment. Figure 6d corresponds to an exemplar raw EMG signal during elbow flexion immediately following the RHD2216 chip while Fig. 6e) shows the filtered data using a [15, 450] Hz bandpass filter.

**Figure 7:**
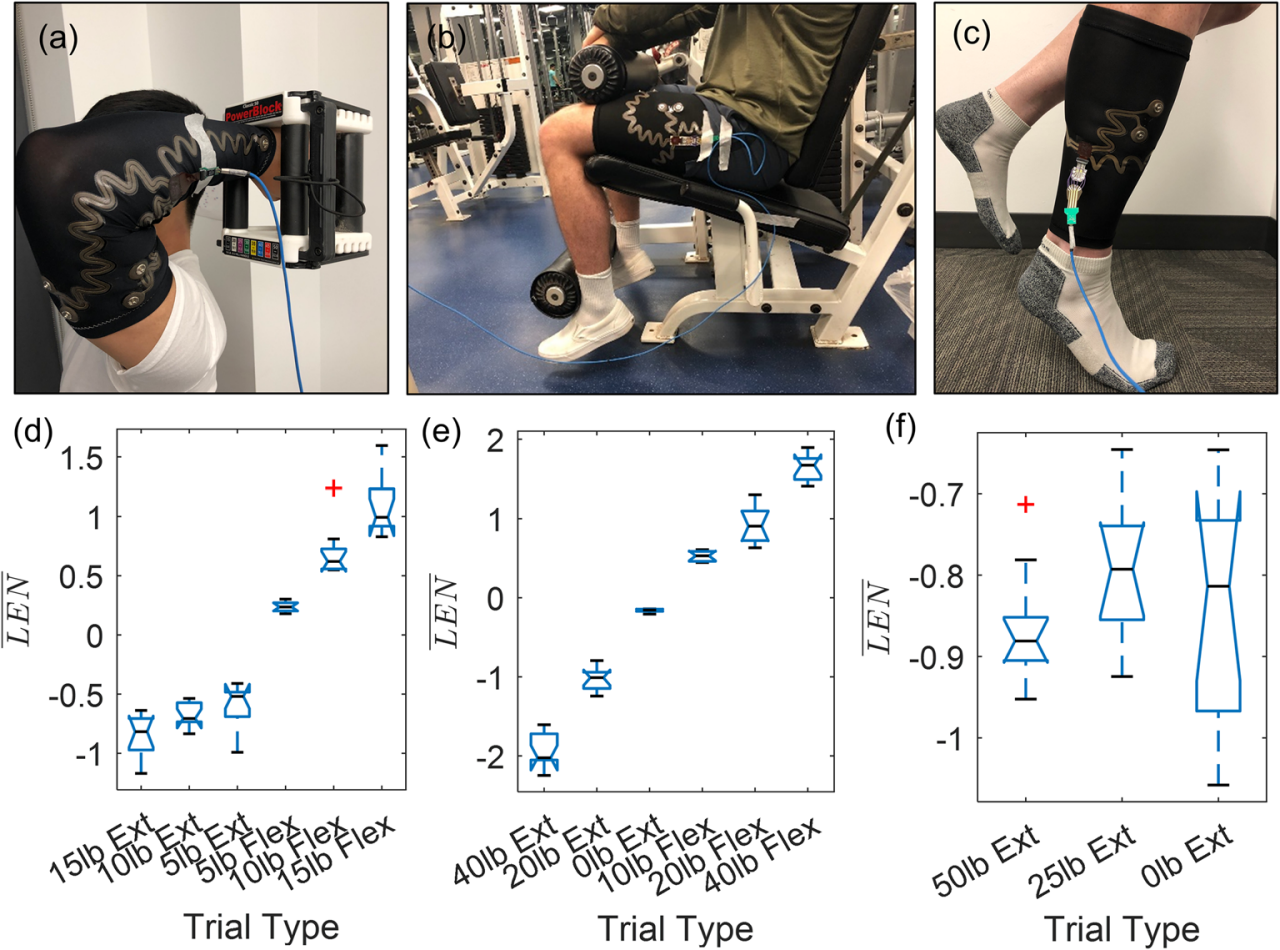
Human subject research testing of sEMG suit with 1 degree of freedom system. Images of experimental set-up for (**a**) the bicep/tricep (elbow), (**b**) quadriceps/hamstring (knee), and (**c**) tibialis anterior/gastrocnemius (ankle) muscle groups (joints). (**d**,**e**) Human subject research data showing 1-way ANOVA results for binned level of effort verification for elbow, knee, and ankle extension exercises, respectively (n = 10 trials). Red crosses indicate outliers.

To demonstrate the efficacy of the finalized sEMG garment, we produced level of effort estimates for the bicep/tricep, quadriceps/hamstring, and tibialis anterior/gastrocnemius muscle groups for human subjects. For these studies, we recorded sEMG signals with the finalized garments while subjects lifted various weights during isolated elbow, knee, and ankle exercises (Fig. 7a–c). Subjects performed single degree of freedom extension exercises for all muscle groups in addition to flexion exercises for the elbow and knee joints. Following data collection, to quantify the variation between muscle activation during weight lifting, we converted raw sEMG data to normalized waveform lengths ($\overline{LEN}$) using maximum voluntary contraction (MVC) measurements and performed a 1-way ANOVA analysis across various weights (Fig. 7d,f).

First, we investigated the efficacy of the arm sleeve. In extension, only the heaviest weight was differentiable from the lightest weight lifted during elbow exercises. Contrastingly, in flexion all three weights at the elbow produced statistically different (p < 0.05) mean $\overline{LEN}$ values compared to the next lowest weight. Additionally, the spread of the data was generally more notable as the weight lifted in flexion increased. This is likely due to an increase in muscle activation during repetitions leading to scatter in the data^38,39^. Further, inconsistent muscle activation and load sharing between superficial and deep muscles, which cannot be recorded by sEMG, may also impact the consistency of sEMG measurements during heavier lifts.

Similarly, at the knee we observed generally increased spread in the data for larger weights lifted in either flexion or extension. However, we were able to differentiate every weight lifted compared to the next lowest weight. Absolute knee $LEN_{Norm}$ values surpassed a value of 1 for the heaviest weights lifted during flexion or extension of the knee, indicating greater muscle activation compared to the static maximum voluntary contractions (MVCs) and consistent with previous studies^40,41^. There was very tight clustering of data during the lightest weight knee extension trial, likely due to the minimal surface muscle activations necessary to perform the knee extension using no added weight.

Of the three joints, the ankle showed the greatest spread in muscle contraction for individual weights, and there was no statistical difference (p > 0.05) between any of the groupings. In the calf sleeve design, we placed only one electrode pair on posterior side of the lower leg, located over the center of the gastrocnemius muscle belly. Previous studies have shown that gastrocnemius activation varies greatly based on distance from origin, as well as between medial and lateral heads^39^. Additionally, deep muscles such as the soleus or muscles of the upper leg which aid in stability, and that were not directly recorded via sEMG, could have inconsistently unloaded the gastrocnemius^42^. Accordingly, the discrepancies reported for the calf sleeve may be addressed by placing the electrode pair more precisely over the preferentially activated regions of the muscle in future designs.

**Figure 8:**
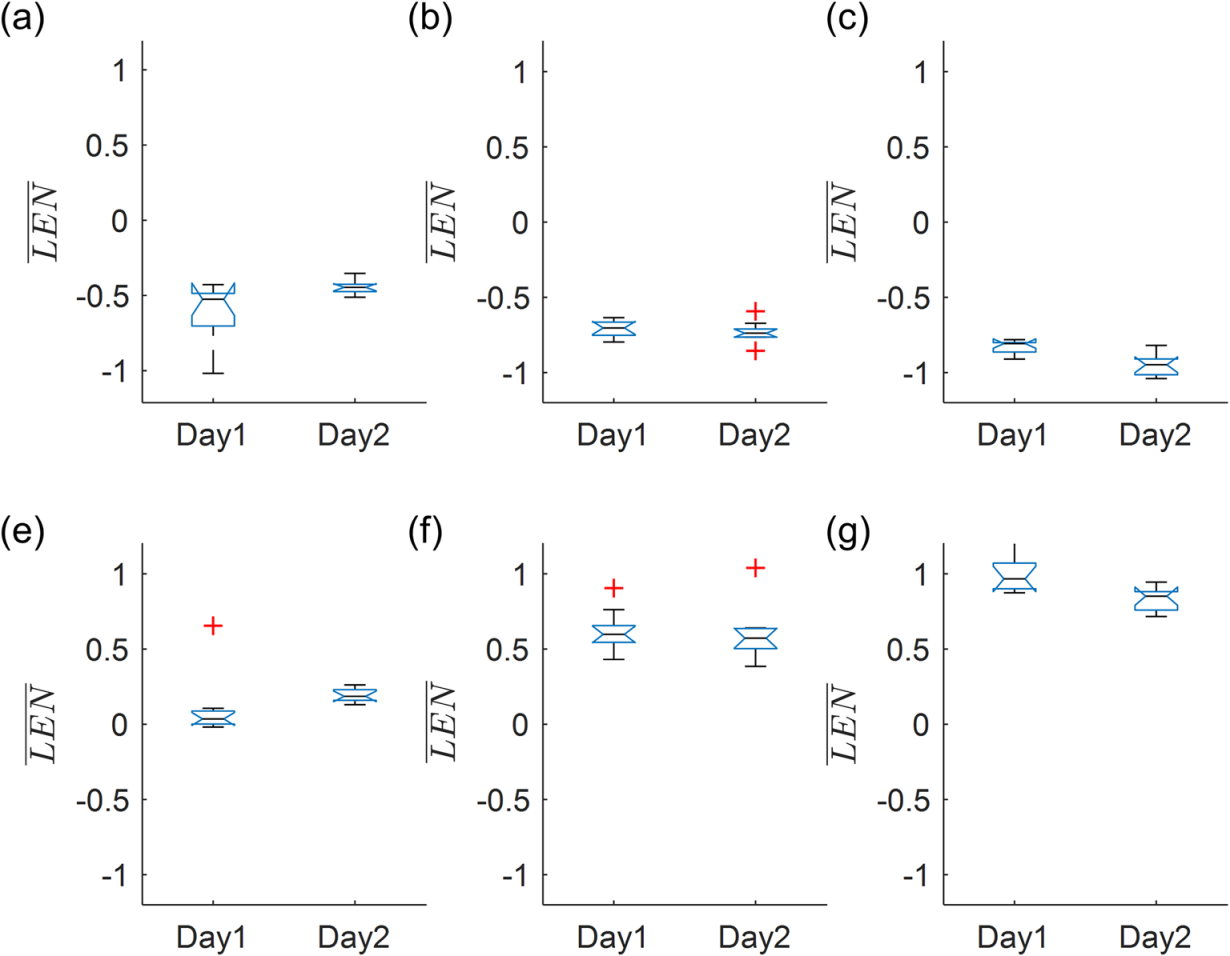
Multi-day comparison of muscle activation during isolated elbow exercises. Red crosses indicate outliers.

Finally, we performed a multi-day comparison of muscle activation during isolated elbow exercises. Notably, these experiments required no recalibration beyond re-calculation of LEN_MVC,_ the muscle activation associated with static MVCs. There was no statistically significant difference (p > 0.05) between the first and second day of trials, for any of the weights lifted in both flexion and extension (Fig. 8). Consequently, relatively little recalibration or electrode position adjustment is necessary in order to obtain consistent results from the garment between multiple sessions.

## Conclusions

In this work, we demonstrated a fully integrated e-textile based sEMG controller for level of effort measurements and characterized its performance. We showed that while screen printed silver ink based interconnects have a lower initial resistance, CCSM dramatically outperformed this material in stability and overall robustness. We demonstrated a marked improvement in the quality of CCSM based leads by optimizing the geometry and overall length such that the end-to-end resistance of each lead was maintained below 200 Ω after 100 don-doff cycles. We found that the laundering process led to a significantly larger increase in resistance for the interconnects compared to the don-doff process, but acceptable resistances were retained through at least 10 machine laundering cycles. In future studies, it may be preferred to hand wash garments as opposed to machine-washing to further extend the lifetime.

In addition to optimizing the interconnects of our sEMG suit, we compared the performance of commercially available CCSM to research grade PEDOT:PSS. We showed that the skin contact impedance of PEDOT:PSS to CCSM is comparable at 1 kHz, and both materials have similar impedances to silver–silver chloride controls when damp. Accordingly, we conclude that textile electrodes function best when either the skin or the textile is slightly damp, to overcome the high impedance of stratum corneum. We achieve this by applying skin moisturizing lotion prior to donning the garment, via dampening the electrodes with water before donning, or through sweat generation during regular use. Notably, this is more easily refreshed than a conventional gel, which ultimately performs the same function for most commercial Ag/AgCl electrodes, and which dry out over time requiring replacement. Abrasion experiments showed comparable performance between CCSM and PEDOT:PSS. The quality of these materials diverged during hand washing, with CCSM outperforming PEDOT:PSS for robustness tests. Thus, being readily commercially available and showing robust electrical stability, CCSM is an ideal electrode material for the sEMG suit. In future studies the performance of the PEDOT:PSS sample can be improved by enhancing the baseline electrical conductivity of PEDOT:PSS^43^ with additives or post-treatment and optimizing the adhesion of the material to an alternative base substrate^44^.

Finally, using a simple, linear, time-domain feature, we demonstrate statistically significant differences between levels of muscle activation measured from the sEMG garment during increasingly difficult exercises. We additionally observed no statistical difference between training sessions. A benefit to this analysis approach is that it requires minimal recalibration between users or sessions. Importantly, this approach is accessible to a relatively uncontrolled operational setting, where computational resources may be low in spite of prediction time constraints. Pattern recognition to detect gross motion was not a focus of this work, but has been demonstrated to high-accuracy using similar time domain features^45^. Future work will investigate the combination of these approaches and extend the number of subjects for a continued assessment of performance and repeatability for intent prediction.

## Materials and methods

### Garment assembly

The baselayer garment was made using a series of laser cutting, alignment, and heat-press lamination steps to create and assemble the textile layers, the 3D printed enclosure, and the Kapton flex tab. Silver ink (PE874, DuPont Intexar) based test coupons and early garment prototypes were patterned with a screen printer (MSP-485, HMI) on single-side adhesive thermoplastic urethane (TPU) films (TE-11C, Dupont Intexar). PEDOT:PSS (655201, 739324, Sigma Aldrich) e-textiles were patterned by dip coating polyester fabric (Spec-Wipe 7,VWR). Stretch athletic base textiles (M-200, Sportek), conductive commercial stretch material (CCSM) (A321, Less EMF), single and double sided (TE-21C, Dupont Intexar) TPU films with integrated melt adhesive, patterned Kapton flex connectors (300 µm thickness, Dupont), and foam cushions (1454120-1540, Online Fabric Store) were cut to appropriate size and shape with a laser-cutter (Fusion Pro 32, Epilog Laser). Nickel plated snap fasteners (116-65, Dritz) were added with a snap application tool (16-P, Dritz). Throughout the process, heat-press steps were performed for 23 s at 290°F unless otherwise noted. For preliminary studies, the stretchable ink was screen printed onto a base TPU layer that was then laser-cut to the desired profile and heat-pressed to the sleeve textile.

First, the CCSM material was heat-press laminated to the exposed surface of double-side adhesive TPU, leaving the paper backer attached on the opposite surface of the TPU for stability during laser cutting and assembly. Next, The CCSM was laser-cut to the desired strain-relief profile for each individual garment piece. Kapton tabs were adhered to the resulting CCSM interconnects using a heat-press and double-sided adhesive TPU to form a low resistance (i.e. ~ 1 Ω) connection. Next, a protective TPU layer was pressed onto the CCSM interconnects and mechanically aligned using pins. Subsequently, the insulated interconnects and Kapton tab assembly was aligned and adhered to the base textile by heat pressing. This completed the garment exterior stack-up. Electrode stack-ups (i.e. cushion, conductive fabric, and TPU) were aligned and attached to the reverse (interior) side of the base textile by heat pressing, again using the double-sided TPU as the adhesive layer. The electrical feedthroughs from the interior to exterior were completed using metal snaps. As a note, the electrodes were spaced 3 cm apart, aligned to the midline of the muscle. A 3 cm spacing was chosen because it is consistent with the smallest possible spacing for the gelled 3 M electrodes used in the comparison studies (see Supplementary Information). Following electrode construction, the interior of the garment was completed by sealing the backside of the custom-built nylon clamshell enclosure into a pocket on the interior of the sleeve by heat pressing with fabric and double-sided TPU layers. The garment was sewn together with a flatlock seam using a serger machine (Victory, Baby Lock) along the laser-cut seam lines, and hem and elastic along the garment top are added for finishing.

Once the garment was completed and donned, the circuit board was attached to the kapton flex tab and the top half of the nylon enclosure was attached. Both halves of the nylon enclosure contained rare-earth magnets glued into recesses to keep the clamshell closed. The top of the enclosure also included locating pins that engage recesses in the enclosure bottom to keep the two halves from sliding apart.

### PEDOT:PSS coupon preparation

PEDOT:PSS solutions were prepared by adapting published protocols^46^. For the preparation of the PEDOT:PSS films, 20 mL of aqueous dispersion (Clevios PH-1000 from Heraeus Holding, details at http://www.clevios.com) were mixed with ethylene glycol (5 mL), dodecyl benzene sulfonic acid (DBSA, 50 μL), and 1 wt% of (3-Glycidyloxypropyl) trimethoxysilane (GOPS), the latter included as a crosslinker to increase stability. Polyester fabric swatches (100% interlock knit polyester fabric from VWR International, Spec-Wipe 7 Wipers) were cut to size and submerged in PEDOT:PSS solution and subjected to ultrasonication for 20 min. Then, the fabric coupon was removed and dried at a temperature of 110 °C. This process was repeated for a total of 4 layers. After the deposition of four layers, the fabric was annealed at 120 °C.

### Tensile testing

Four different interconnect shapes (n = 3 for each shape) were compared using waveform cyclic testing conducted on an Instron Load Frame. Samples were run with a frequency of 1 Hz and amplitude displacement cycled between 0 and 25% Strain for 1000 cycles. During testing, the resistance of the attached sample was measured using a voltage divider, applying a constant voltage to the textile interconnect and a monitoring resistor connected in series, and measuring the voltage across the resistor at the maximum and minimum strain point of each cycle. The resistances plotted in Fig. 3 correspond to the maximum strain point, which was approximately 10% higher than the values at the minimum strain point.

### Laundering cycles

For machine washing, the circuit board was removed from the Kapton tab and stored separately in an ESD safe bag or box so as not to damage the electronics. The garment was then placed inside a delicates bag and washed on delicate cycle, with liquid detergent (YShield, Texcare), using water temperature no higher than 40 °C. Once complete, the garment was air dried to avoid softening and sticking of the TPU. Resistance was measured only after the garment had fully dried.

For hand washing, fabric coupons were washed in a detergent/water solution (0.1 vol. % YShield, Texcase) for a 30 s agitation followed by a 30 min soak. After soaking, samples were removed from the washing solution, wrung, and dabbed dry with a kimwipe. Samples were left overnight to dry and sheet resistance was measured the following morning.

### Impedance measurements

A Gamry Reference 3000 Electrochemical Impedance Analyzer was used to monitor impedance in a symmetric two-electrode configuration, with the electrodes positioned on the bicep of a healthy male volunteer. Using the Potentiostatic Electrochemical Impedance measurement protocol, the frequency was swept from 1 to 10,000 Hz, at 10 steps a decade, using an AC voltage of 25 mV, and a baseline DC voltage of 0 V, and impedance between the two electrodes was collected. This measurement included two skin/electrode interfaces to complete the measurement circuit, so the impedance of a single skin/electrode interface was approximately one-half of the values plotted in Fig. 5d. Prior to measurement, the system was allowed to stabilize for 10 s before measurements began. Values of real impedance versus frequency were plotted for subsequent analysis.

### Simulated UV exposure

Conductive textiles were subject to simulated accelerated weathering environment using Q-Panel QUV Accelerated Weathering Tester set to1.1 W/m^2^/nm UVA-340 peak and approximately 1.75 × intensity of full sun at 55 °C. The total exposure duration was approximately 1 week, but samples were removed ~ 3 h prior to each measurement to allow for stabilization and account for real changes caused by thermal expansion/contraction. The total exposure was 153.25 h, having an equivalent exposure (UVA-340 peak) of 268 h.

### Abrasion studies

Wet textile coupons were assessed for abrasion resistance using a Taber Linear Abraser. In the test, a 1 kgf load was applied to a 1 cm probe covered with a transfer cloth. The cloth covered probe was programmed to travel back and forth along an approximately 5.1 cm path length at 1 Hz. Following tests, sheet resistance was measured and qualitative visual observations were made.

### Electronics assembly

The electronics assembly was composed of a Kapton flex tab, a custom designed printed circuit board (PCB), sealing gasket, and custom glass filled nylon enclosure. 300 µm thick polyimide flex tabs were fabricated following standard flex PCB procedures using ½ oz. copper and plated with electroless nickel/immersion gold (ENIG).

The PCB consisted primarily of an electrophysiology amplifier chip (RHD2216, INTAN technologies), FPC socket (FH52, Hirose), and SPI connector for serial communication with the processing board (C3100, INTAN technologies) or, in other versions, a 32-bit embedded microcontroller (EFM32, Giant Gecko). The amplifier received, amplified, and digitized the sEMG signals collected by the garment. The embedded system interfaced with the garment via the FPC socket, and additionally performed the on-board level of effort analysis.

The enclosure served as the protection for the electronics from dust, impact, and water. It was a custom-designed printed clamshell made from glass filled nylon beads (PA 3200 GF, EOS) using selective laser sintering (P395, EOS). Magnets embedded in the plastic clamshell provided the closing force and sealing pressure to the TPU gasket. A circular hole at one end allowed the RS485 serial cable to connect the embedded system inside the enclosure to external systems outside the enclosure.

### Human subject research study

Written informed consent was obtained from all subjects and/or their legal guardian(s) in accordance with the relevant guidelines and regulations. All experiments and protocols involving human participants were reviewed and approved by the Johns Hopkins Medicine Institutional Review Board (IRB). The experimental protocol involved three healthy subjects (males, 28 ± 2 years) who were selected to perform isolated exercises with a single degree of freedom; either the elbow, knee, or ankle (Table 1), while wearing the arm sleeve, shorts, or leg sleeve garments, respectively. Subjects were screened to ensure a proper garment fit for the garment size. Each subject was introduced to the exercises for the joint under test and was asked to self-select three distinct weights corresponding to their comfort level performing a small (3), medium (10), or large (20) number of repetitions. Skin at the electrode sites was prepared with rubbing alcohol and lotion before the garment was donned; after donning, the garment was checked by the experimenter for proper placement on the agonist and antagonist muscle groups, but was not readjusted or removed during data collection. One subject returned for a second day of elbow data collection in order to compare performance consistency. Data were collected in accordance with ethical approvals by the Johns Hopkins Medicine Institutional Review Board under protocol IRB00170676.

**Table 1:** Exercises, along with agonist and antagonist muscle groups.

Joint under test	Extension exercise	Flexion exercise	Agonist muscle groups (extension/flexion)
Elbow	Overhead triceps dumbbell extension, neutral grip	Biceps dumbbell curl, neutral grip	Triceps/biceps
Knee	Seated leg extension (cable)	Seated hamstring curl (cable)	Quadriceps/hamstring
Ankle	Standing calf raise while holding dumbells	N/A	Gastrocnemius/tibialis anterior

Exercises performed by the subjects included: holding a static maximum voluntary contraction (MVC) for both flexion and extension of the joint under test, as well as ten sets of a single repetition of extension exercises at each weight. MVC was repeated several times throughout the course of data collection. Flexion exercises (ten sets at each weight) were performed only for the elbow and knee joints.

A simple measure for each subject’s level of effort during each exercise repetition was calculated from the raw sEMG data. sEMG data was filtered using a 4-pole bandpass filter with lower and upper cutoff frequencies of 20 Hz and 400 Hz, respectively^47^. For each electrode pair, the waveform length ($LEN_{i}$) was calculated (Eq. 1) using a 20 ms sliding window with a slide-size of 5 ms and a sample size of N^48^.

1 $$LEN_{i} = \mathop \sum \limits_{k = 1}^{N} \left| {\Delta x_{k} } \right|;\quad where \quad \Delta x_{k} = x_{k} - x_{k - 1}$$

$LEN_{i}$ values for antagonist muscle groups were discarded, and the mean agonist curve length feature ($\overline{LEN}$) was calculated by averaging across each agonist sEMG channel for each sample (Eq. 2). The peak absolute value of $\overline{LEN}$ during the concentric portion of each movement was recorded, and normalized by the average peak of the mean curve length feature during the MVC trials for the corresponding degree of freedom and motion direction (LEN_MVC_) (Eq. 3). Separate LEN_MVC_ values were calculated for each day of testing for the subject who returned multiple times. The normalized value $LEN_{Norm}$ was set to positive in flexion, and negative in extension, and represents the peak concentric muscle activation during each trial.

2 $$\overline{LEN} = \mathop \sum \limits_{i} LEN_{i}$$

3 $$LEN_{Norm} = \frac{{{\text{max}}\left( {\left| {\overline{LEN} } \right|} \right)}}{{{\text{max}}\left( {\left| {\overline{{LEN_{MVC} }} } \right|} \right)}}$$

A one-way analysis of variance (ANOVA) was conducted (Matlab, Mathworks Inc.) to determine statistically significant differences between $LEN_{Norm}$ during single-joint repetitions of varying intensity, based on amount of weight lifted. Additionally, ANOVA was conducted to determine variation between days for the one subject who was tested on two separate occasions.

## Supplementary Information

Supplementary Information.

## References

[1] Samuel OW (2019). Intelligent EMG pattern recognition control method for upper-limb multifunctional prostheses: Advances, current challenges, and future prospects. IEEE Access.

[2] Schweisfurth MA (2016). Electrotactile EMG feedback improves the control of prosthesis grasping force. J. Neural Eng..

[3] Adewuyi AA, Hargrove LJ, Kuiken TA (2016). Evaluating EMG feature and classifier selection for application to partial-hand prosthesis control. Front. Neurorobot..

[4] Nyni, K. A. *et al.* Wireless health monitoring system for ECG, EMG and EEG detecting. in *2017 International Conference on Innovations in Information, Embedded and Communication Systems *(*ICIIECS*) 1–5. 10.1109/ICIIECS.2017.8275879 (2017).

[5] Liu S-H (2019). An EMG patch for the real-time monitoring of muscle-fatigue conditions during exercise. Sensors.

[6] Finni T, Hu M, Kettunen P, Vilavuo T, Cheng S (2007). Measurement of EMG activity with textile electrodes embedded into clothing. Physiol. Meas..

[7] Zhang J, Wang B, Zhang C, Xiao Y, Wang MY (2019). An EEG/EMG/EOG-based multimodal human-machine interface to real-time control of a soft robot hand. Front. Neurorobot..

[8] Ferreira A (2008). Human-machine interfaces based on EMG and EEG applied to robotic systems. J. Neuroeng. Rehabil..

[9] Moin A (2021). A wearable biosensing system with in-sensor adaptive machine learning for hand gesture recognition. Nat. Electron..

[10] Lee S, Kim M-O, Kang T, Park J, Choi Y (2018). Knit band sensor for myoelectric control of surface EMG-based prosthetic hand. IEEE Sens. J..

[11] Pino, E. J., Arias, Y. & Aqueveque, P. Wearable EMG shirt for upper limb training. in *2018 40th Annual International Conference of the IEEE Engineering in Medicine and Biology Society *(*EMBC*) 4406–4409. 10.1109/EMBC.2018.8513107 (2018). 10.1109/EMBC.2018.8513107 30441329

[12] Guo, J. *et al.* A soft robotic exo-sheath using fabric EMG sensing for hand rehabilitation and assistance. in *2018 IEEE International Conference on Soft Robotics *(*RoboSoft*) 497–503. 10.1109/ROBOSOFT.2018.8405375 (2018).

[13] Zhang, R., Bernhart, S. & Amft, O. Diet eyeglasses: Recognising food chewing using EMG and smart eyeglasses. in *2016 IEEE 13th International Conference on Wearable and Implantable Body Sensor Networks *(*BSN*) 7–12. 10.1109/BSN.2016.7516224 (2016).

[14] Farina, D., Lorrain, T., Negro, F. & Jiang, N. High-density EMG E-Textile systems for the control of active prostheses—IEEE Conference Publication. https://ieeexplore.ieee.org/abstract/document/5627455 (2010). 10.1109/IEMBS.2010.5627455 21096838

[15] Zeng W (2014). Fiber-based wearable electronics: A review of materials, fabrication, devices, and applications. Adv. Mater..

[16] Wang C (2019). Advanced carbon for flexible and wearable electronics. Adv. Mater..

[17] Wang B, Facchetti A (2019). Mechanically flexible conductors for stretchable and wearable E-skin and E-textile devices. Adv. Mater..

[18] Andrew TL (2018). Melding vapor-phase organic chemistry and textile manufacturing to produce wearable electronics. Acc. Chem. Res..

[19] Agcayazi T, Chatterjee K, Bozkurt A, Ghosh TK (2018). Flexible interconnects for electronic textiles. Adv. Mater. Technol..

[20] Ling Y (2020). Disruptive, soft, wearable sensors. Adv. Mater..

[21] Jin H (2017). Enhancing the performance of stretchable conductors for E-textiles by controlled ink permeation. Adv. Mater..

[22] Pani D (2019). Validation of polymer-based screen-printed textile electrodes for surface EMG detection. IEEE Trans. Neural Syst. Rehabil. Eng..

[23] Qu J (2019). Screen printing of graphene oxide patterns onto viscose nonwovens with tunable penetration depth and electrical conductivity. ACS Appl. Mater. Interfaces.

[24] Guo R (2019). Semiliquid metal enabled highly conductive wearable electronics for smart fabrics. ACS Appl. Mater. Interfaces.

[25] La T-G (2018). Two-layered and stretchable E-textile patches for wearable healthcare electronics. Adv. Healthc. Mater..

[26] Atalay O, Atalay A, Gafford J, Walsh C (2018). A highly sensitive capacitive-based soft pressure sensor based on a conductive fabric and a microporous dielectric layer. Adv. Mater. Technol..

[27] Cheng T, Zhang Y, Lai W-Y, Huang W (2015). Stretchable thin-film electrodes for flexible electronics with high deformability and stretchability. Adv. Mater..

[28] Liu Y, He K, Chen G, Leow WR, Chen X (2017). Nature-inspired structural materials for flexible electronic devices. Chem. Rev..

[29] Zhang Y (2013). Buckling in serpentine microstructures and applications in elastomer-supported ultra-stretchable electronics with high areal coverage. Soft Matter.

[30] Afroj S, Tan S, Abdelkader AM, Novoselov KS, Karim N (2020). Highly conductive, scalable, and machine washable graphene-based E-textiles for multifunctional wearable electronic applications. Adv. Funct. Mater..

[31] Gaubert V, Gidik H, Bodart N, Koncar V (2020). Investigating the impact of washing cycles on silver-plated textile electrodes: A complete study. Sensors.

[32] uz Zaman S, Tao X, Cochrane C, Koncar V (2019). Launderability of conductive polymer yarns used for connections of E-textile modules: Mechanical stresses. Fibers Polym..

[33] Sadeqi A (2018). Washable smart threads for strain sensing fabrics. IEEE Sens. J..

[34] Tseghai GB, Mengistie DA, Malengier B, Fante KA, Van Langenhove L (2020). PEDOT:PSS-based conductive textiles and their applications. Sensors.

[35] Rossetti N (2019). Poly(3,4-ethylenedioxythiophene) (PEDOT) coatings for high-quality electromyography recording. ACS Appl. Bio Mater..

[36] Sinha SK (2017). Screen-printed PEDOT:PSS electrodes on commercial finished textiles for electrocardiography. ACS Appl. Mater. Interfaces.

[37] Kee S (2019). Highly stretchable and air-stable PEDOT:PSS/ionic liquid composites for efficient organic thermoelectrics. Chem. Mater..

[38] Yasuda T, Brechue WF, Fujita T, Sato Y, Abe T (2008). Muscle activation during low-intensity muscle contractions with varying levels of external limb compression. J. Sports Sci. Med..

[39] Kinugasa R, Kawakami Y, Fukunaga T (2005). Muscle activation and its distribution within human triceps surae muscles. J. Appl. Physiol..

[40] Masuda K, Masuda T, Sadoyama T, Inaki M, Katsuta S (1999). Changes in surface EMG parameters during static and dynamic fatiguing contractions. J. Electromyogr. Kinesiol..

[41] Schipplein OD, Andriacchi TP (1991). Interaction between active and passive knee stabilizers during level walking. J. Orthop. Res..

[42] Narici MV (1996). Human quadriceps cross-sectional area, torque and neural activation during 6 months strength training. Acta Physiol. Scand..

[43] Mengistie DA, Ibrahem MA, Wang P-C, Chu C-W (2014). Highly conductive PEDOT:PSS treated with formic acid for ITO-free polymer solar cells. ACS Appl. Mater. Interfaces.

[44] Ryan JD, Mengistie DA, Gabrielsson R, Lund A, Müller C (2017). Machine-washable PEDOT:PSS dyed silk yarns for electronic textiles. ACS Appl. Mater. Interfaces.

[45] Zardoshti-Kermani M, Wheeler BC, Badie K, Hashemi RM (1995). EMG feature evaluation for movement control of upper extremity prostheses. IEEE Trans. Rehabil. Eng..

[46] Sessolo M (2013). Easy-to-fabricate conducting polymer microelectrode arrays. Adv. Mater..

[47] Stegeman, D. F. & Hermens, H. J. Standards for surface electromyography: The European project "Surface EMG for non-invasive assessment of muscles (SENIAM)” 108–112 (1998).

[48] Tkach D, Huang H, Kuiken TA (2010). Study of stability of time-domain features for electromyographic pattern recognition. J. Neuroeng. Rehabil..

