# Supplementary material for: E-textile based modular sEMG suit for large area level of effort analysis
Source: Sci Rep. 2022 Jun 10;12:9650. doi: 10.1038/s41598-022-13701-4 (PMC9187645; doi:10.1038/s41598-022-13701-4)
Supplement: Supplementary file 1 — Supplementary Information. [file 41598_2022_13701_MOESM1_ESM.pdf]

Supplementary Information

E-Textile Based Modular sEMG Suit for Large Area Level of Effort Analysis

Korine A. Ohiri[1], Connor O. Pyles[1], Leslie H. Hamilton[1], Megan M. Baker[1], Matthew T. McGuire[1], Eric Q. Nguyen[1], Luke E. Osborn[1], Katelyn M. Rossick[1], Emil G. McDowell[1], Leah M. Strohsnitter[2], Luke J. Currano[1,*]

[1] Research and Exploratory Development Department, The Johns Hopkins Applied Physics Laboratory, Laurel, MD 20723, USA

[2] Air and Missile Defense Sector, The Johns Hopkins Applied Physics Laboratory, Laurel, MD 20723, USA

*__Corresponding Author:__ Luke.Currano@jhuapl.edu

Garment Selection

The Sportek M-200 garment is composed of 92% polyester and 8% spandex and thus is advertised to exhibit excellent stretchability and wicking ability/ moisture management. For our end application we needed a garment that was lightweight, exhibited excellent wicking performance, and was desired to be fire resistant. To ensure this fabric indeed excels in these areas, we compared the M-200 against seven others by Sportek (i.e. FM-60/FM-65, SP-VX5000, P-600, F-JX200, F-56/F-78, and SP-XP52, F-27060) along with one by RockyWoods (under armour). Wicking was determined by immersing the bottom of a test coupon into a water bath and tracking the water height over time (Fig. S1).

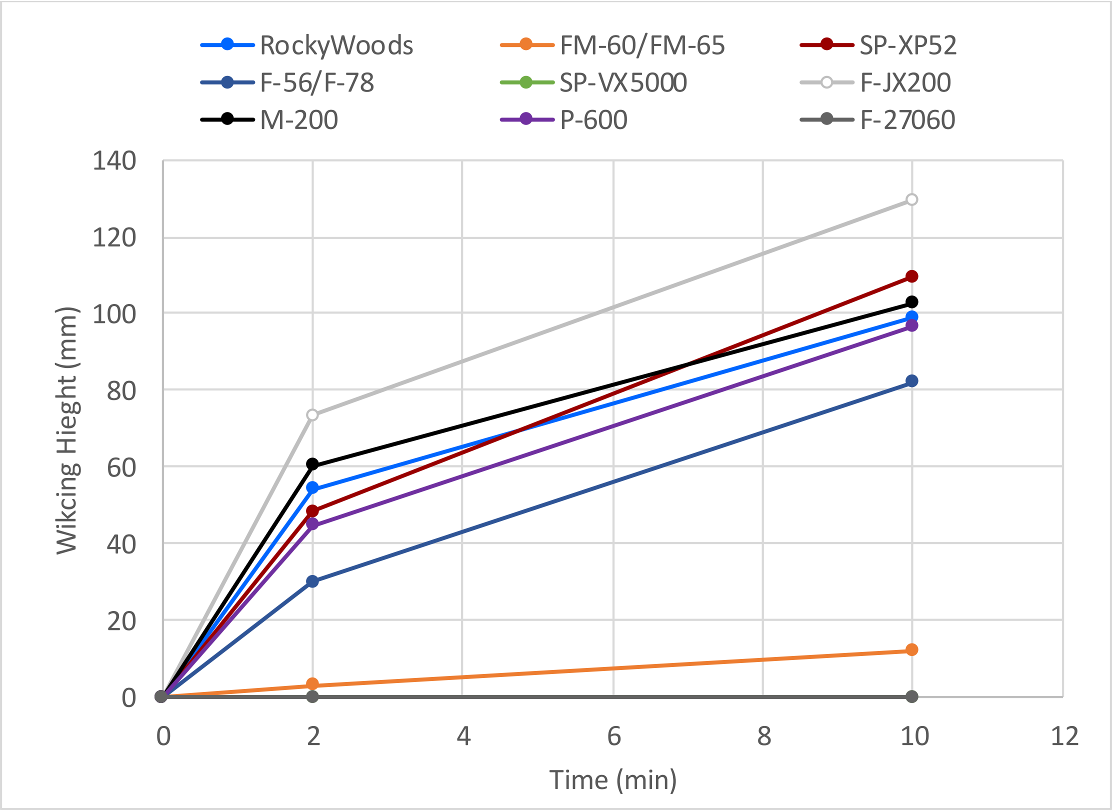

Figure S1. Wicking height versus time for all nine fabrics

From this it is clear that F-JX200, SP-XP52, and M-200 exhibit the best wicking performance over time. These three fabrics have a normalized weight (g/m^2) of 200, 296, and 244 respectively, thus we eliminated the SP-XP52 from consideration for being too heavyweight. Finally, although the F-JX200 material showed promise for wicking performance and weight, it is comprised of 15% Spandex and 85 % Nylon. While neither material burns easily, polyester melts at a slightly higher temperature than nylon (i.e. ~250°C compared to 220°C). As a result, M-200 was selected as our final material.

Baseline Noise Analysis

Baseline noise was calculated by analyzing the sEMG signal during rest (e.g., no movement) while the arm garment was being worn by a user. Six subjects were recruited to don the arm garment and position the electrodes over the bicep, lateral tricep, and long tricep muscle groups. Disposable Ag-AgCl gel electrodes (3M) placed on the same location as the sleeve electrodes to enable comparison of the different electrode materials from across multiple trials. A 1 s window of the sEMG signal during the no movement period was segmented for this analysis. The root mean square of the middle 50% of the data was calculate to produce the noise levels during rest for each electrode type. Across the six participants, there were 27 trials with data from the Ag-AgCl gel electrodes, 24 trials from the CCSM electrodes, and 49 trials from the PEDOT electrodes. Statistical analysis between the electrode groups was performed using a two-sample t-test.

To ensure quality of the sEMG garment and compare across different electrode materials, we recorded baseline noise levels of the arm sleeve while being worn by a user (Fig. S2). To compare with commercially available sEMG electrodes, we manually placed disposable Ag-AgCl gel electrodes on the same muscle groups targeted by the arm garment. The user was not moving while data was collected to capture the baseline noise levels. We compared multiple sleeves (n=6) to also verify the suitability of CCSM electrodes for sEMG recordings. Overall, the CCSM electrode material showed slightly less noise levels compared to a standard Ag-AgCl commercial electrode ($p < 0.01$ at two locations). The PEDOT electrode material showed slightly higher noise levels, but not statistically significant ($p > 0.05$), compared to the Ag-AgCl gel electrodes. Overall, both CCSM and PEDOT electrode garments were within acceptable ranges for sEMG recordings during gross movements.

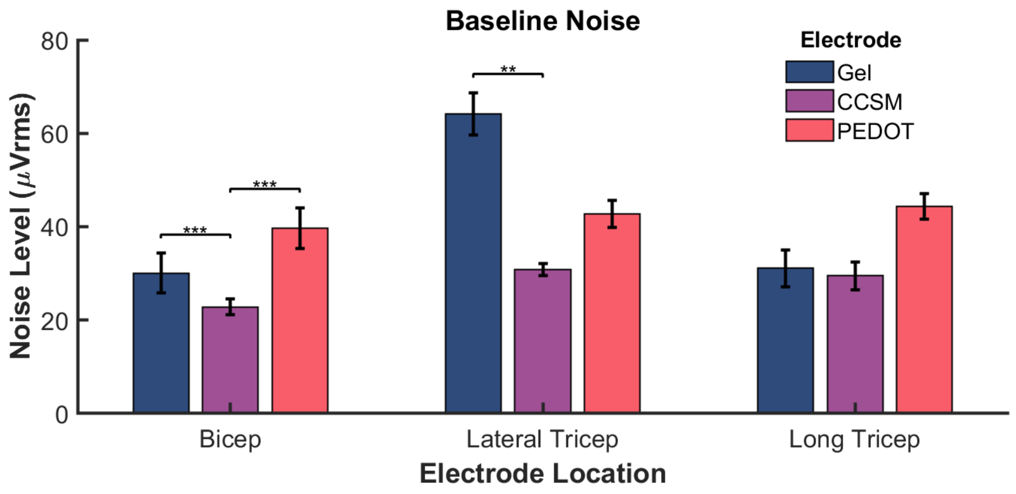

Figure S2. Baseline sEMG noise levels of CCSM and PEDOT electrodes. Arm garments with either CCSM or PEDOT electrodes were worn by a user at rest while baseline signal noise levels were recorded from targeted muscle sites. Compared to standard Ag-AgCl gel electrodes, CCSM electrodes had lower noise levels ($p < 0.01$ at

two locations) whereas the PEDOT electrode garment showed similar noise levels ($p > 0.05$) to the Ag-AgCl gel electrodes.

Electrode Signal-to-Noise Ratio

SNR was calculated by taking the middle 50% of a 1 s segment from sEMG signals recorded during arm flexion or extension and taking the ratio of that movement signal to baseline sEMG recordings (i.e., during no limb movement). Six participants were recruited to perform arm flexion and extension movements while recording sEMG signals. Arm garments with different electrode materials (i.e., CCSM and PEDOT) were used to compare across material type. Participants also performed the arm movements with disposable Ag-AgCl gel electrodes placed on the same muscle groups targeted by the arm garments. SNR for each movement type (i.e., flexion and extension) was calculated and results were combined across participants based on electrode type. Across the six participants, there were 27 trials with data from the Ag-AgCl gel electrodes, 24 trials from the CCSM electrodes, and 49 trials from the PEDOT electrodes. Statistical analysis between the electrode groups was performed using a two-sample t-test.

To determine usability, we calculated the signal-to-noise ratio (SNR) of the garments while being worn by a user during flexion and extension arm movements (Fig. S3). SNR was calculated for each muscle and compared across the different electrode types. In all cases, SNR for the CCSM electrode garment was greater than the commercial Ag-AgCl gel electrode, and was over the accepted threshold of 1.2 dB for usable sEMG

signals in movement decoding tasks.[1] The PEDOT electrode garment was also above the acceptable SNR threshold, but was generally lower than the CCSM electrode garment. These results demonstrate the ability of the textile-based garments to capture reliable and usable sEMG signals.

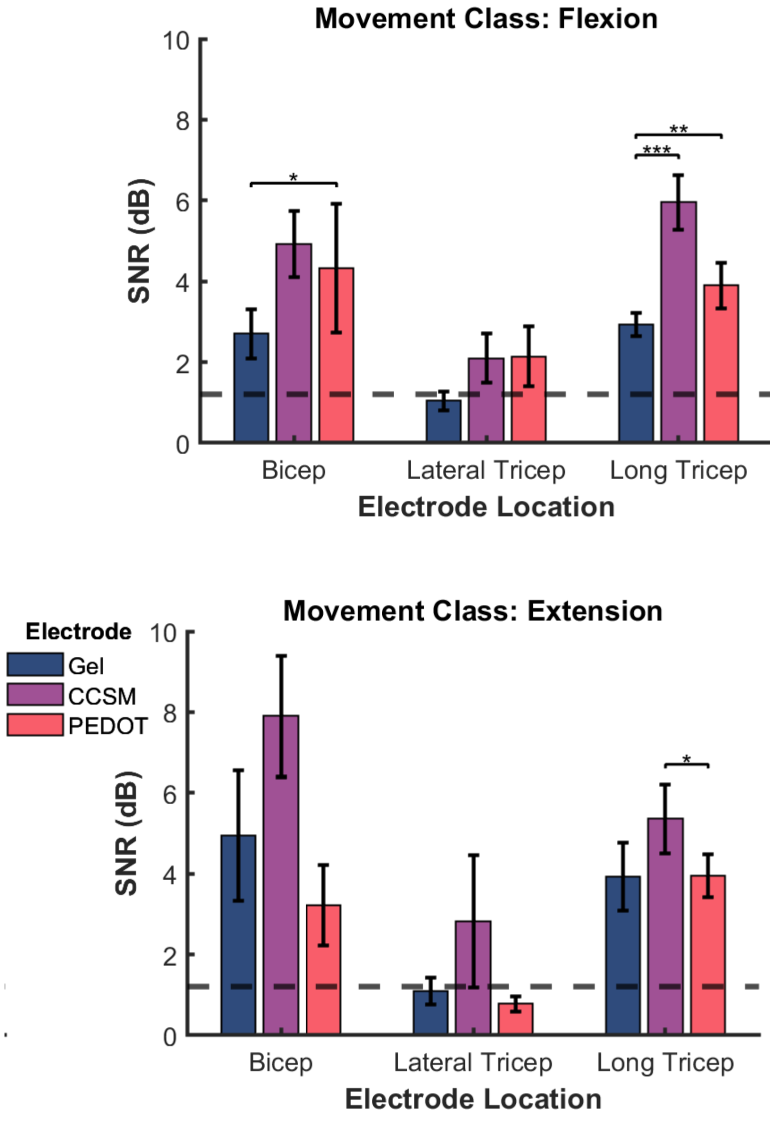

Figure S3. Electrode sEMG SNR during arm movement. A user wore arm garments with different electrode materials and performed arm flexion and extension movements. The CCSM and PEDOT electrode types generally outperformed standard Ag-AgCl

electrode performance for each muscle group location. In all cases, the CCSM electrodes were above the 1.2 dB threshold for usable sEMG signals during movement.

References

[1] Delsys. 2020. How To Improve EMG Signal Quality – Signal Quality Monitor - Delsys. [online] Available at: <https://delsys.com/emgworks/signal-quality-monitor/improve/> [Accessed 29 September 2020].
